# Ultra-processed food intake, diet quality, and risk of gestational diabetes mellitus: a cross-sectional analysis from the Mutaba’ah study

**DOI:** 10.1186/s12986-025-00950-z

**Published:** 2025-05-26

**Authors:** Aisha A. Almulla, Hanna Augustin, Luai A. Ahmed, Linnea Bärebring

**Affiliations:** 1 Department of Internal Medicine and Clinical Nutrition, Institute of Medicine, Sahlgrenska Academy, University of Gothenburg, Gothenburg, Sweden; 2 Dietary Services, Tawam Hospital, Abu Dhabi Health Services Company (SEHA), Abu Dhabi, United Arab Emirates; 3 Institute of Public Health, College of Medicine and Health Sciences, United Arab Emirates University, Al Ain, United Arab Emirates; 4 Zayed Centre for Health Sciences, United Arab Emirates University, Al Ain, United Arab Emirates

**Keywords:** Ultra-Processed foods, Pregnancy, Gestational diabetes mellitus, Diet quality, Mediterranean diet

## Abstract

**Background:**

High intake of Ultra-Processed Foods (UPF) has raised concerns about how they might impact maternal diet and potentially increase the risk of Gestational Diabetes Mellitus (GDM). This study aimed to evaluate the associations between UPF intake or adherence to the Mediterranean Diet and GDM among pregnant women in the United Arab Emirates.

**Methods:**

Pregnant women (*n* = 1054) from the dietary subcohort within the prospective Mutaba’ah Study cohort were included. Diet was assessed through a semi-quantitative food frequency questionnaire, and UPF intake in servings/day was classified according to the NOVA system. The alternate Mediterranean Diet (aMED) score specific for pregnancy defined adherence to the Mediterranean Diet. GDM diagnosis was based on the National Institute for Health and Clinical Excellence criteria. Logistic regression models adjusted for maternal age, first trimester body mass index, parity, gestational age, education level, employment status, physical activity, and husband’s smoking status were used to assess associations between UPF intake or aMED score and GDM.

**Results:**

Mean ± SD UPF intake was 9.4 ± 3.4 servings/day and mean aMED score was 4.0 ± 1.5. Women in the highest tertile of UPF intake had lower aMED score than those in the lowest tertile (4.3 ± 1.4 vs. 3.6 ± 1.4, *P* < 0.001). Women in the highest tertile of UPF intake had higher intakes of carbohydrates, saturated fatty acids, sodium, and selenium than those in the lowest tertile, while intakes of protein, total fat, monounsaturated fatty acids, and most micronutrients were lower (*P* < 0.05). Neither tertiles of UPF intake (third tertile compared to the lowest OR = 0.85, 95% CI: 0.54–1.34) nor continuous UPF intake (OR = 0.97, 95% CI: 0.92–1.03) was associated with GDM. Similarly, aMED score was not associated with GDM in either tertile of the score (third tertile compared to the lowest OR = 0.94, 95% CI: 0.54–1.64) or as a continuous variable (OR = 0.99, 95% CI: 0.87–1.11).

**Conclusions:**

Higher intake of UPF was associated with a lower adherence to the Mediterranean Diet. However, neither UPF intake nor aMED score was associated with GDM.

**Supplementary Information:**

The online version contains supplementary material available at 10.1186/s12986-025-00950-z.

## Introduction

The International Diabetes Federation estimated that in 2019, the global prevalence of diabetes in pregnancy was 15.5%, with Gestational Diabetes Mellitus (GDM) making up 12.8% [1]. The prevalence of GDM in the United Arab Emirates (UAE) has been reported at 27% among Emirati women, reflecting a high burden of GDM in this population [2]. The critical role of nutrition during pregnancy in promoting maternal and child health is well-established and widely recognized [3, 4]. Therefore, a high intake of Ultra-Processed Foods (UPF) among pregnant women may be of concern due to the increased nutritional needs of both the mother and the developing fetus. UPF are industrially formulated products containing minimal whole foods and typically include additives such as flavorings, colorings, preservatives, and emulsifiers to enhance taste, texture, and shelf-life [5, 6]. These foods are classified under the NOVA classification system that categorizes foods based on processing level, dividing them into four groups: (1) unprocessed or minimally processed foods, (2) processed culinary ingredients, (3) processed foods, and (4) UPF [5]. UPF are often nutrient-poor and energy-dense [6], and contributes substantially to energy intake in the United States [7] and Europe [8]. High consumption of UPF has been associated with various negative health outcomes in adults, including an increased risk of obesity, cardiovascular diseases, type 2 diabetes, and all-cause mortality [9–11].

Research indicates that high UPF consumption among pregnant women is associated with a lower intake of vegetables, fruit, protein, fiber, vitamins, and minerals [12, 13]. Studies regarding associations between UPF intake and pregnancy outcomes, such as GDM, show mixed results [14–17]. Pregnancy induces a state of insulin resistance [18], which may be exacerbated by a diet high in UPF, potentially leading to GDM [16]. In addition, healthy dietary patterns during pregnancy, including the Mediterranean Dietary pattern, could decrease the risk of GDM [19, 20]. The Mediterranean Diet emphasizes fruits, vegetables, legumes, whole grains, and foods high in Monounsaturated Fatty Acids (MUFA), components that may contribute to the potential preventive effects of the Mediterranean diet against GDM [21].

The UAE, with its multicultural population and diverse food environment, has undergone substantial dietary shifts, leading to increased consumption of processed and high-fat foods [22, 23]. These changes reflect a broader global trend where greater food availability erodes traditional diets, gradually replacing them with more Westernized diets [24]. This shift poses health concerns for pregnant women, as high UPF intake is associated with inadequate nutrient intake and an increased risk of pregnancy complications, including GDM. Currently, no study has examined the association between diet and GDM in the UAE, highlighting a regional gap in knowledge. Therefore, this study aims to evaluate the association between UPF intake or adherence to the Mediterranean Diet and GDM among pregnant women in the UAE.

## Materials and methods

### Study design and setting

Data used in these analyses are drawn from the Mutaba’ah Study [25], an ongoing prospective cohort study aiming to investigate early life determinants of maternal, infant, child, and adolescent health in the UAE. Recruitment started in May 2017, including pregnant women from the Emirati population who were 18 years or older, residents of Al Ain, and who could provide informed consent. More detailed information about the Mutaba’ah Study has been published previously [25]. Women recruited between the 9th of December 2019 and the 26th of August 2022 were asked to provide dietary data, and those who accepted formed the dietary subcohort within the Mutaba’ah Study. In the dietary subcohort, a total of 1531 pregnant women were enrolled. In the current analysis, women with implausible reported energy intake (< 600 kcal (*n* = 31) or > 4000 kcal/day (*n* = 378)) [26, 27], multifetal pregnancy (*n* = 63), and preexisting diabetes (*n* = 5) were excluded. Ethical approval was obtained from the UAE University Human Research Ethics Committee (ERH-2017-5512) and the Abu Dhabi Health Research and Technology Ethics Committee (DOH/CVDC/2022/72) [25]. Informed written consent was obtained from all participants prior to data collection. All study procedures were performed according to the guidelines of the Declaration of Helsinki.

Study data were obtained from medical records and questionnaires [25]. At inclusion to the Mutaba’ah Study, a baseline self-administered questionnaire was administered to obtain data on maternal demographic characteristics, including maternal age, gestational age, parity (categorized as nulliparous/multiparous), university-level education (categorized as yes/no), employment status (categorized as employed/not employed), husband’s smoking status (defined as husband smoking during the current pregnancy and categorized as yes/no), and physical activity before pregnancy (reporting any physical activity at least once a week before pregnancy and was categorized as yes/no). Imputation of missing values was performed by replacing the missing responses with the most common (mode) answer regarding the husband’s smoking status and physical activity before pregnancy. First trimester (gestational week ≤ 14) weight and height were extracted from medical records, and Body Mass Index (BMI) was calculated (kg/m^2^) and categorized based on the criteria from the World Health Organization [28].

### Dietary data collection and processing

Dietary intake was assessed using a validated, self-administered 146-item semi-quantitative Food Frequency Questionnaire (FFQ) at recruitment to the Mutaba’ah Study during one of the antenatal care visits at any time point during pregnancy. Thus, dietary data were collected during all three trimesters. In the FFQ, women were asked to choose from nine possible intake frequencies, ranging from never or less than once/month to > 6 times/day. Imputation was applied for missing responses to individual FFQ items, mainly applied in the sweets and baked goods category, by replacing the missing values with the means of the available responses for that specific item. Detailed information about the FFQ has been published previously [29].

UPF intake was classified according to the NOVA system [6, 30]. A detailed account of how classifications were made is shown in Supplementary File 1. Due to limited information on processing techniques for some traditional and region-specific foods, their classification within the NOVA system required careful assessment. To address this, we considered the most consumed products in the market and double-checked the food labels and package information to ensure accurate classification. UPF intake was defined as servings per day, and energy adjusted using the residual method [31].

The alternate Mediterranean diet (aMED) score [32–34] was used to evaluate the overall dietary quality of the women and has been modified for pregnancy by excluding alcohol intake [35]. The aMED is based on eight components: vegetables, fruits, nuts, whole grains, legumes, fish, Monounsaturated Fatty Acids to Saturated Fatty Acids (MUFA/SFA) ratio, and red and processed meat. Components were energy adjusted and standardized to 2000 kcal per day. The aMED score is based on the median intake for each component within the study sample. For the healthy components (vegetables, legumes, fruits, nuts, whole grains, fish, and the ratio of MUFA/SFA), intake equal to or above the median was scored as 1 point, and intake below the median was scored as 0 points. For red and processed meat intake, the scoring was reversed. Thus, the total score ranged from zero to eight, with a higher score representing higher adherence to the Mediterranean diet. The aMED score has been previously described elsewhere [34–36].

### Gestational diabetes mellitus (GDM)

All pregnant women were recommended to undergo GDM screening between 24 and 28 weeks of pregnancy using a 75-gram 2-hour Oral Glucose Tolerance Test (OGTT) [2]. Women were instructed to be fasting for at least 8 h before the OGTT, and glucose levels were measured in fasting blood and after 1- and 2-hour post-load. GDM diagnosis in the current analyses was based on the National Institute for Health and Clinical Excellence (NICE 2015) criteria. The NICE 2015 cutoff points [37, 38] were set as fasting glucose ≥ 5.6 mmol/L or 2-h glucose ≥ 7.8 mmol/L. GDM was defined as at least one glucose value equal to or above the cutoff.

### Statistical analysis

Analyses were reported based on the guidelines supported by Strengthening the Reporting of Observational Studies in Nutritional Epidemiology (STROBE-nut) [39]. Data were described as means and Standard Deviations (SD) for continuous variables (mean ± SD) or frequencies and proportions for categorical variables. Normality was examined by histograms. Independent variables were tested for collinearity by Spearman correlation. Energy adjusted UPF intake in servings/day and aMED score were categorized into tertiles.

Differences in maternal characteristics (maternal age, gestational age at the time of the FFQ completion, first trimester BMI, parity, university level education, employment status, husband’s smoking status, and physical activity before pregnancy) according to UPF intake were tested using chi-square for categorical variables, and Kruskal-Wallis for continuous variables. For first trimester BMI, underweight (BMI < 18.5) was grouped with normal weight (BMI < 25) due to few women in the underweight group (*N* = 31). The association between UPF intake with aMED score and intake of nutrients and food groups were assessed using Mann-Whitney U test, comparing low (tertile 1) to high (tertile 3) UPF intake. Logistic regression analysis was used to assess the association between total UPF intake (both as a continuous variable and tertiles of intake) and intake of UPF groups (dairy, mixed dishes, bread, sweets, and beverages) with GDM diagnosis. Logistic regression was also used to investigate associations between aMED score (both as a continuous variable and tertiles of intake) and its components (vegetables (100 g/d), fruits (100 g/d), nuts (30 g/d), whole grains (30 g/d), legumes (100 g/d), fish (100 g/d), MUFA/SFA ratio (g/d), and red and processed meat (100 g/d)) with GDM diagnosis. These analyses were presented as three models: Model (1) crude, unadjusted, model (2) adjusted for maternal age, first trimester BMI, parity, gestational age at the time of the FFQ, education level, employment status, physical activity before pregnancy, husband’s smoking status and model (3) additionally adjusted for either aMED score or UPF intake. The lowest tertile was considered as the reference category in the analysis. The selected covariates in the models were identified according to prior knowledge and by a Directed Acyclic Graph [40]. Adjusted Odds Ratios (OR) with their 95% Confidence Intervals (CI) were derived from the univariable and multivariable models. A sensitivity analysis was conducted to evaluate the robustness of the study results, in which we included only women who completed the FFQ before week 24 of pregnancy (i.e., including only women who had answered the FFQ before the time of GDM screening). A p-value of less than 0.05 was considered statistically significant for all analyses. Statistical analyses were conducted using IBM SPSS Statistics for Windows, version 28.0 (IBM Corporation).

## Results

### UPF intake and maternal characteristics

Characteristics of the pregnant women and according to UPF intake are shown in Table 1. Women with higher UPF intake were significantly younger, nulliparous, unemployed, had husbands who more often smoked, and reported not engaging in physical activity before pregnancy (*P* < 0.05). Women with normal/underweight in the first trimester had higher UPF intake than obese women (*p* = 0.04).

**Table 1:** Association between UPF intake and maternal characteristics of pregnant women

Maternal characteristics	Mean ± SD	UPF servings/day					
	Overall	Tertile 1	Tertile 2	Tertile 3	*P*-value^1^	Total *N*	
Age, years	31 ± 6	32.6 ± 6.1	29.7 ± 5.8	29.9 ± 5.8	< 0.001	1054	
Gestational age, months	6.5 ± 2.1	6.6 ± 2.0	6.5 ± 2.1	6.4 ± 2.2	0.82	1054	
First trimester BMI, kg/m^2^	27.2 ± 5.8	27.9 ± 6.1	26.4 ± 5.0	27.2 ± 6.2	0.051	793	
	**N (%)**						
Trimester of pregnancy					0.15		
1st trimester	123 (12)	29 (23.6)	46 (37.4)	48 (39.0)			
2nd trimester	316 (30)	115 (36.4)	99 (31.3)	102 (32.3)			
3rd trimester	615 (58)	207 (33.7)	207 (33.7)	201 (32.7)			
First trimester BMI, kg/m^2^					0.07		
Under- and normal weight < 25	299 (37.7)	91 (30.4)	111 (37.1)	97 (32.4)			
Overweight 25-29.9	272 (34.3)	106 (39.0)	89 (32.7)	77 (28.3)	0.101*		
Obese ≥ 30	222 (28.0)	86 (38.7)	61 (27.5)	75 (33.8)	0.044*		
Parity					< 0.001	1054	
Nulliparous	300 (28.5)	73 (24.3)	117 (39.0)	110 (36.7)			
Multiparous	754 (71.5)	278 (36.9)	235 (31.2)	241 (32.0)			
University level education					0.19	985	
No	511 (52)	155 (30.3)	179 (35.0)	177 (34.6)			
Yes	474 (48)	169 (35.7)	149 (31.4)	156 (32.9)			
Employment status					0.014	982	
Employed	284 (29)	112 (39.4)	80 (28.2)	92 (32.4)			
Unemployed	698 (71)	211 (30.2)	246 (35.2)	241 (34.5)			
Husband smoking					0.005	1054	
No	686 (65.1)	252 (36.7)	221 (32.2)	213 (31.0)			
Yes	368 (34.9)	99 (26.9)	131 (35.6)	138 (37.5)			
Physical activity before pregnancy					0.021	1054	
No	802 (76.1)	249 (31.0)	278 (34.7)	275 (34.3)			
Yes	252 (23.9)	102 (40.5)	74 (29.4)	76 (30.2)			

BMI: body mass index, SD: standard deviation, UPF: ultra-processed food

^1^Kruskal-Wallis test for continuous variables and chi-square test for categorical variables. *Compared to under- and normal-weight women

### UPF intake and diet quality

**Fig. 1:**
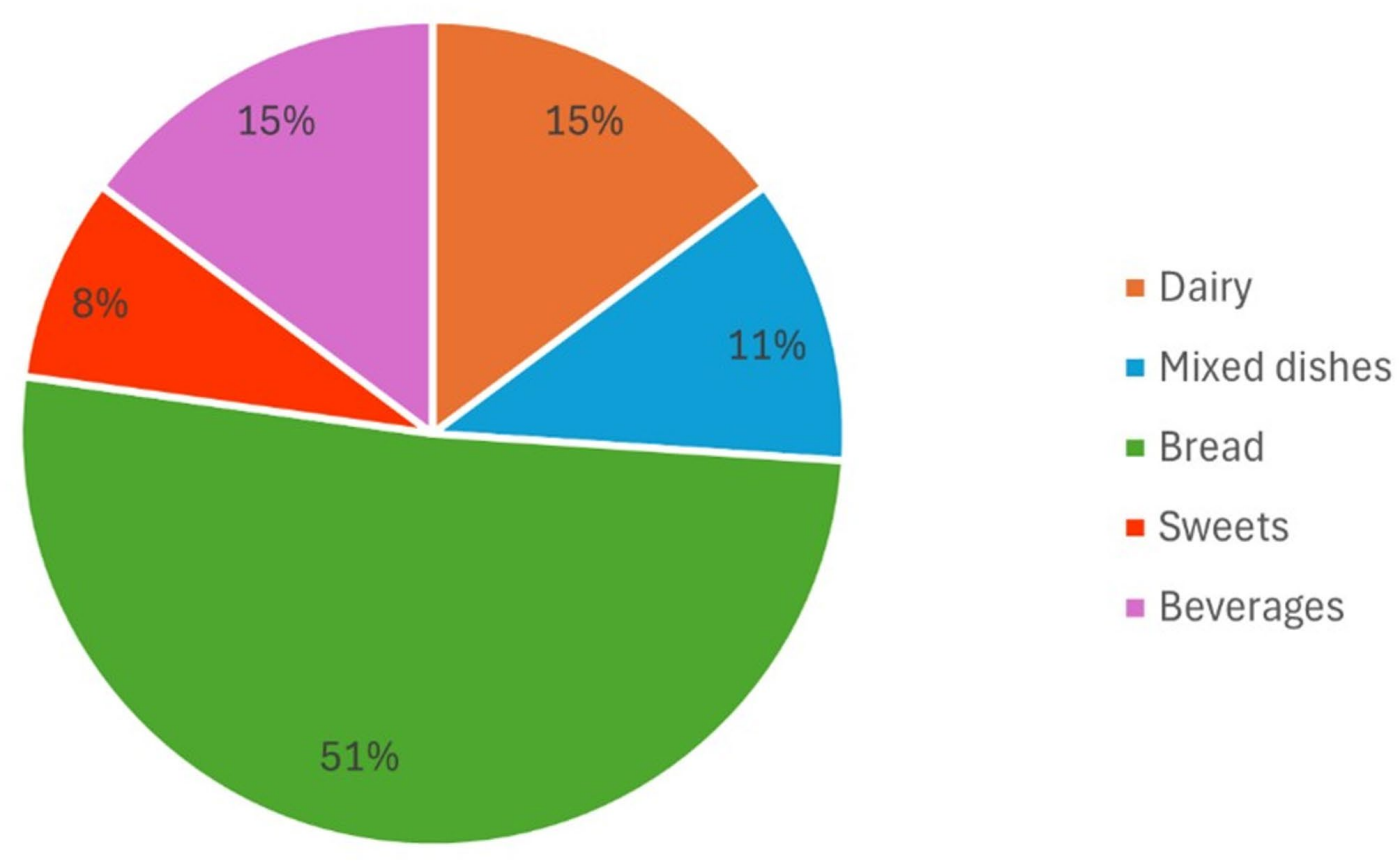
Energy percent contribution of UPF food groups to total UPF energy intake

The mean daily energy intake was 2237 ± 814 kcal (Table 2). UPF intake provided 26 ± 10% of total energy intake with a mean intake of 9.4 ± 3.4 servings/day. The median and interquartile ranges (IQR) of UPF intake was 9.2 servings/day (IQR: 7.4–11.1). The largest contributor to energy among UPF food groups was bread (51 E%), followed by dairy (15 E%), beverages (15 E%), mixed dishes (11 E%), and sweets (8 E%) (Fig. 1.).

aMED score was significantly lower in the highest tertile of UPF intake compared to the lowest tertile (Table 2). Intake of carbohydrates and Saturated Fatty Acids (SFA) was higher in the highest tertile of UPF intake, while intake of protein, total fat, and MUFA was lower compared to those in the lowest tertile. Intake of almost all micronutrients was significantly lower among women in the highest tertile of UPF intake compared to women in the lowest tertile, except for higher intakes of sodium and selenium. For food groups, those in the highest tertile of UPF intake had a lower intake of vegetables, fruit, red meat, legumes, and nuts but a higher intake of whole grain and sweetened beverages compared to the lowest tertile (Table 2).

**Table 2:** Association between UPF intake and dietary nutrient intake and food groups of pregnant women

Nutritional profile		UPF servings/day energy adjusted, Mean ± SD			*P*-value*
	Overall	Tertile 1	Tertile 2	Tertile 3	
UPF intake (servings/day)	9.4 ± 3.4	6.1 ± 1.8	9.3 ± 0.7	12.9 ± 2.8	< 0.001
aMED score	4.02 ± 1.5	4.3 ± 1.4	4.2 ± 1.5	3.6 ± 1.4	< 0.001
Total energy intake (kcal/d)	2236.6 ± 814	2338 ± 881	2026 ± 750	2347 ± 766	0.796
Carbohydrate intake (E%)	49.4 ± 8.9	49.1 ± 10.5	48.7 ± 7.9	50.5 ± 7.9	0.003
Protein intake (E%)	16.2 ± 3.3	16.7 ± 3.7	16.7 ± 3.1	15.2 ± 2.9	< 0.001
Fat intake (E%)	37.4 ± 6.9	38.1 ± 8.2	37.4 ± 6.3	36.7 ± 6.3	0.009
SFA intake (E%)	12.2 ± 3.1	11.8 ± 3.1	12.4 ± 3.2	12.4 ± 3.0	0.002
MUFA intake (E%)	14.7 ± 3.4	15.5 ± 4.2	14.7 ± 2.7	14.0 ± 2.8	< 0.001
PUFA intake (E%)	6.9 ± 2.4	7.3 ± 3.2	6.7 ± 1.8	6.6 ± 1.9	0.067
Fiber (g/1000 kcal)	12.7 ± 3.6	14.6 ± 4.0	12.2 ± 3.1	11.4 ± 2.9	< 0.001
Iron (mg/1000 kcal)	7.1 ± 1.4	7.2 ± 1.5	6.9 ± 1.4	6.9 ± 1.4	< 0.001
Calcium (mg/1000 kcal)	379.8 ± 154.7	397.9 ± 177.7	377.1 ± 148.0	364.4 ± 133.9	0.002
Magnesium (mg/1000 kcal)	173.9 ± 33.1	193.6 ± 36.1	170.5 ± 25.8	157.9 ± 25.9	< 0.001
Potassium (mg/1000 kcal)	1639.5 ± 290.7	1791.7 ± 319.9	1622.9 ± 232.8	1503.9 ± 235.7	< 0.001
Sodium (mg/1000 kcal)	1037.4 ± 331.3	970.0 ± 385.9	1077.5 ± 325.5	1064.6 ± 261.1	< 0.001
Zinc (mg/1000 kcal)	5.0 ± 1.2	5.4 ± 1.4	5.0 ± 0.9	4.7 ± 1.1	< 0.001
Selenium (µg/1000 kcal)	52.0 ± 12.3	49.8 ± 13.2	54.6 ± 11.9	51.8 ± 11.4	0.028
Vitamin D (µg/1000 kcal)	1.7 ± 1.2	1.7 ± 1.2	1.8 ± 1.3	1.7 ± 0.9	0.882
Retinol equivalents (µg/1000 kcal)	3297.6 ± 1803.2	3945.3 ± 2179.8	3130.9 ± 1578.6	2817.1 ± 1360.9	< 0.001
Beta-carotene (µg/1000 kcal)	1338.4 ± 864.7	1661.9 ± 1031.1	1266 ± 738.1	1087.1 ± 683.3	< 0.001
Vitamin C (mg/1000 kcal)	7.1 ± 1.4	95.2 ± 49.8	82.6 ± 39.9	75.9 ± 35.9	< 0.001
Vitamin E (mg/1000 kcal)	4.7 ± 1.8	5.4 ± 2.3	4.5 ± 1.4	4.3 ± 1.5	< 0.001
Vitamin B12 (µg/1000 kcal)	4.1 ± 3.2	4.5 ± 3.9	4.2 ± 2.9	3.5 ± 2.4	< 0.001
Folate (µg/1000 kcal)	224.3 ± 54.8	233.2 ± 60.7	220.0 ± 51.5	219.7 ± 50.9	0.004
Vegetables (g/d/1000 kcal)	56.5 ± 48.4	67.2 ± 53.2	55.1 ± 45.9	47.2 ± 43.5	< 0.001
Fruits (g/d/1000 kcal)	240.2 ± 153.8	311.4 ± 183.1	223.2 ± 130.6	185.9 ± 110.4	< 0.001
Total diary (g/d/1000 kcal)	134.6 ± 122.7	135.1 ± 143.9	137.3 ± 120.1	131.3 ± 100.3	0.198
Low fat dairy (g/d/1000 kcal)	23.9 ± 62.5	29.9 ± 78.6	21.3 ± 55.8	20.4 ± 48.9	0.170
Wholegrain (g/d/1000 kcal)	12.8 ± 13.4	10.8 ± 12.3	13.2 ± 12.7	14.5 ± 15.0	< 0.001
White meat (g/d/1000 kcal)	41.9 ± 26.7	40.3 ± 27.6	48.5 ± 28.7	37.1 ± 22.1	0.458
Fish (g/d/1000 kcal)	20.4 ± 20.2	20.1 ± 22.8	23.1 ± 20.4	17.9 ± 16.8	0.166
Red meat (g/d/1000 kcal)	15.8 ± 14.5	17.6 ± 17.9	16.4 ± 11.8	13.4 ± 12.9	< 0.001
Legumes (g/d/1000 kcal)	12.7 ± 16.5	15.9 ± 21.7	11.6 ± 11.7	10.6 ± 14.1	< 0.001
Nuts (g/d/1000 kcal)	15.8 ± 17.9	22.1 ± 23.9	13.6 ± 13.2	11.8 ± 12.5	< 0.001
Sweetened beverages (g/d/1000 kcal)	216.4 ± 196.4	139.7 ± 137.2	213.7 ± 130.6	295.7 ± 260.6	< 0.001

* Mann-Whitney U tests (for the comparison between low and high tertiles)

UPF: ultra-processed food, SD: standard deviation, aMED: alternate Mediterranean Diet score, SFA: Saturated fatty acids, MUFA: Monounsaturated fatty acids, PUFA: polyunsaturated fatty acids, E%: % of total energy/day

### UPF intake, aMED score, and GDM

The incidence of GDM was 25.2%. There were no significant associations between total UPF intake expressed as energy adjusted servings/day (presented as a continuous variable and categorical in tertiles) or UPF food groups and GDM in the regression analyses (Table 3). In addition, there were no significant associations between aMED score (presented as a continuous variable and categorical in tertiles) or its components and GDM (Table 4). The results of the sensitivity analyses among women who had answered the FFQ before the time of GDM screening were consistent with the main findings (Supplementary File 2). In addition, an exploratory first trimester BMI-stratified analysis showed no significant associations with total UPF intake or aMED score, suggesting the robustness of our findings across different BMI categories (data not shown).

**Table 3:** UPF intake according to GDM diagnosis defined by the NICE-2015 criteria*

	OR	95% CI	*P* value
**UPF intake (continuous, servings/day)**			
Model 1, crude	0.98	0.94–1.02	0.37
Model 2, adjusted^1^	0.98	0.93–1.03	0.36
Model 3, adjusted^2^	0.97	0.92–1.03	0.34
**UPF intake (tertiles**,** servings/day)**			
Model 1, crude			
Tertile 1	Ref		
Tertile 2	0.92	0.65–1.32	0.66
Tertile 3	0.85	0.59–1.21	0.36
Model 2, adjusted^1^			
Tertile 1	Ref		
Tertile 2	0.97	0.63–1.51	0.89
Tertile 3	0.86	0.55–1.34	0.49
Model 3, adjusted^2^			
Tertile 1	Ref		
Tertile 2	0.97	0.63–1.51	0.89
Tertile 3	0.85	0.54–1.34	0.48
**UPF food groups**			
**UPF dairy (servings/day)**			
Model 1, crude	1.14	1.00-1.31	0.050
Model 2, adjusted^1^	1.15	0.97–1.36	0.10
Model 3, adjusted^2^	1.16	0.98–1.39	0.092
**UPF mixed dishes (servings/day)**			
Model 1, crude	1.06	0.82–1.36	0.68
Model 2, adjusted^1^	0.99	0.73–1.36	0.99
Model 3, adjusted^2^	0.99	0.73–1.36	0.99
**UPF bread (servings/day)**			
Model 1, crude	0.97	0.85–1.11	0.64
Model 2, adjusted^1^	0.96	0.81–1.13	0.59
Model 3, adjusted^2^	0.96	0.81–1.12	0.59
**UPF sweets (servings/day)**			
Model 1, crude	0.94	0.76–1.17	0.57
Model 2, adjusted^1^	0.89	0.67–1.18	0.40
Model 3, adjusted^2^	0.89	0.67–1.18	0.40
**UPF beverages (servings/day)**			
Model 1, crude	0.85	0.70–1.03	0.091
Model 2, adjusted^1^	0.83	0.64–1.07	0.14
Model 3, adjusted^2^	0.82	0.64–1.07	0.14

UPF: ultra-processed food, GDM: gestational diabetes mellitus, OR: odds ratio, CI: confidence intervals, Ref: reference, NICE-2015: National Institute for Health and Clinical Excellence. ^1^Adjusted for maternal age, first trimester BMI, parity, gestational age at the time of the FFQ, education level, occupation status, physical activity before pregnancy, and husband smoking. ^2^Adjusted for model two and aMED score. * *N* = 965 in the crude model and *N* = 686 in the adjusted models

**Table 4:** aMED score according to GDM diagnosis defined by the NICE-2015 criteria*

	OR	95% CI	*P* value
**aMED score (continuous)**			
Model 1, crude	1.06	0.96–1.16	0.29
Model 2, adjusted^1^	0.99	0.88–1.12	0.96
Model 3, adjusted^2^	0.99	0.87–1.11	0.82
**aMED score (tertiles)**			
Model 1, crude			
Tertile 1	Ref		
Tertile 2	1.31	0.95–1.82	0.09
Tertile 3	1.25	0.81–1.94	0.31
Model 2, adjusted^1^			
Tertile 1	Ref		
Tertile 2	1.24	0.83–1.85	0.29
Tertile 3	0.97	0.56–1.68	0.92
Model 3, adjusted^2^			
Tertile 1	Ref		
Tertile 2	1.21	0.81–1.81	0.35
Tertile 3	0.94	0.54–1.64	0.83
**aMED components**			
**Vegetable (100 g/d)**			
Model 1, crude	0.99	0.86–1.17	0.99
Model 2, adjusted^1^	0.99	0.83–1.19	0.97
Model 3, adjusted^2^	0.98	0.82–1.18	0.86
**Fruits (100 g/d)**			
Model 1, crude	0.99	0.95–1.04	0.79
Model 2, adjusted^1^	0.99	0.93–1.05	0.65
Model 3, adjusted^2^	0.97	0.92–1.04	0.40
**Nuts (30 g/d)**			
Model 1, crude	1.05	0.93–1.18	0.43
Model 2, adjusted^1^	1.01	0.87–1.18	0.88
Model 3, adjusted^2^	0.99	0.85–1.16	0.95
**Whole grain (30 g/d)**			
Model 1, crude	1.09	0.93–1.28	0.29
Model 2, adjusted^1^	0.93	0.75–1.14	0.48
Model 3, adjusted^2^	0.94	0.76–1.17	0.58
**Legumes (100 g/d)**			
Model 1, crude	0.98	0.63–1.54	0.94
Model 2, adjusted^1^	0.82	0.48–1.39	0.47
Model 3, adjusted^2^	0.79	0.46–1.36	0.39
**Red meat (100 g/d)**			
Model 1, crude	0.83	0.49–1.42	0.50
Model 2, adjusted^1^	0.93	0.51–1.69	0.80
Model 3, adjusted^2^	0.89	0.48–1.65	0.72
**Fish (100 g/d)**			
Model 1, crude	1.11	0.79–1.57	0.55
Model 2, adjusted^1^	1.19	0.74–1.89	0.47
Model 3, adjusted^2^	1.18	0.74–1.89	0.49
**MUFA/SFA ratio (g/d)**			
Model 1, crude	1.07	0.86–1.35	0.54
Model 2, adjusted^1^	1.01	0.77–1.33	0.96
Model 3, adjusted^2^	0.99	0.75–1.31	0.94

aMED: alternate Mediterranean Diet score, GDM: gestational diabetes mellitus, MUFA/SFA: monounsaturated fatty acids to saturated fatty acids, OR: odds ratio, CI: confidence intervals, Ref: reference, NICE-2015: National Institute for Health and Clinical Excellence.^1^Adjusted for maternal age, first trimester BMI, parity, gestational age at the time of the FFQ, education level, occupation status, physical activity before pregnancy, and husband smoking. ^2^Adjusted for model two and UPF. * *N* = 965 in the crude model and *N* = 686 in the adjusted models

## Discussion

The results of this cohort study showed that a higher intake of UPF was associated with an overall negative impact on diet quality, indicated by a lower aMED score. However, our findings showed no association between UPF intake or aMED and GDM.

In our study, UPF intake accounted for 26% of total energy intake, aligning closely with findings from a cohort study of pregnant women in Brazil (25.5%) [41] and Spain (29.7%) [16]. In contrast, higher proportions have been observed in the United States (52.6%) [12] and lower levels in Italy (about 10%) [42]. To our knowledge, there are no previous data on UPF intake in UAE populations. This substantial variability in the percentage of total energy intake from UPF across countries can be attributed to differences in UPF intake measurement and definition, dietary patterns, food availability, cultural preferences, and economic factors. We also found lower diet quality, indicated by the aMED score, with an increasing UPF intake. Our results align with evidence that higher UPF intake, particularly in the upper tertiles or quartiles, is linked to lower adherence to a Mediterranean diet in pregnant women, adults, and older populations [43–45]. Results from our study further showed that higher UPF intake was negatively associated with nutrient intake and related to intake of food groups which is consistent with previous studies [12, 13]. The type of foods contributing to UPF intake varies among countries. According to data from 80 countries, the most consumed UPF included baked goods, dairy products, and, among beverages, carbonated drinks [46]. The largest contributor to energy among UPF food groups in our study was bread, which might explain why pregnant women with higher UPF intake had higher carbohydrate and whole grain intake. Recent studies indicate that bread and whole grain foods, despite many being classified as UPF, do not appear to have detrimental implications for health [47, 48]. However, the suggested negative impact of UPF on nutrient intake in our study is concerning, as pregnancy is a period when nutritional requirements are markedly increased [49].

We used the NOVA classification system to classify UPF intake, which classifies foods high in added sugars, saturated fat, and sodium as UPF but also foods with a healthier nutrient profile [5, 6, 50]. While the NOVA classification system is widely used to identify UPF, its application in epidemiological studies is still evolving, which may explain inconsistencies between studies due to variations in classification methods and data collection approaches [6, 51]. Additionally, diet assessment methods used in most research do not specify the level of food processing, which can lead to misclassification of foods [52]. Based on the different classification criteria used to define UPF, high UPF consumption during pregnancy is linked to mixed results in its association with increasing the risk of GDM. In this study, no association was found between UPF intake and GDM. Similar to our results, a cross-sectional study among Brazilian pregnant women found no significant association between high UPF intake and GDM [17]. Further, systematic reviews have found no evidence that high UPF intake, classified by NOVA, during pregnancy is associated with risk of GDM. However, the systematic reviews found that intake of some UPF subtypes such as fast food and soft drinks is associated with GDM [14, 15]. A possible explanation for these somewhat disparate findings might be due to the broad classification of food as UPF according to NOVA. Fast food and soft drinks have unfavorable nutrient profiles and that is a likely explanation for the observed associations of higher intake of these foods and GDM risk. In contrast, many whole grain food items in our study were classified as UPF and previous research has found that higher consumption of whole grain is associated with a decreased risk of GDM diagnosis [53]. Thus, the NOVA broad UPF category likely does not capture the complexity of a healthy diet. Furthermore, some researchers argue that NOVA’s broad categorizations may oversimplify food quality, leading to inconsistent diet-health associations across studies [54, 55]. These results underscore the complexity of the relationship between UPF intake with varying definitions and categorization across studies and GDM risk, emphasizing the need for more nuanced research to understand the role of UPF in pregnancy-related health outcomes fully. Further research with more precise dietary assessment tools is necessary to explore the effects of UPF intake on health during pregnancy, as well as the potential mechanisms underlying these associations and the extent to which these are the direct effect of food processing level or via confounding factors (e.g., poor diet quality, sedentary lifestyle, socioeconomic factors, education level, depression etc.). Exploring alternative methods for UPF classification is warranted for future investigation. Future research should aim to standardize UPF definitions and classifications to enable more robust and comparable findings.

Although we found no association between UPF intake and GDM, UPF consumption during pregnancy may have other significant clinical implications due to its adverse effects on diet quality. Adequate energy and nutrient intake during pregnancy is crucial, as it directly influences Gestational Weight Gain (GWG) and birth weight [56]. Increased energy intake from UPF is associated with greater GWG and neonatal adiposity [57], which may be relevant in the UAE, where the incidence of excessive GWG is high [58]. Therefore, high intake of some types of UPF, including fast foods, processed meat, and sugary snacks, should be discouraged during pregnancy, while an increased intake of minimally processed, whole foods with a healthy nutrient profile should be promoted [14]. Healthcare providers, including dietitians, should provide personalized guidance, regular assessments, and connect patients with appropriate resources to support healthy eating habits throughout pregnancy. Moreover, combining traditional foods with modern nutritional knowledge can align cultural practices with health-conscious choices.

Results from previous studies are also mixed regarding the association between healthy dietary patterns during pregnancy and the risk of GDM [59]. For example, a study in 10 Mediterranean countries found that greater adherence to a Mediterranean dietary pattern was inversely associated with the risk of GDM and associated with improved glucose tolerance [60]. Moreover, in a United States cohort of pregnant women, higher aMED scores during preconception and pregnancy were related to a lower risk of GDM [35]. Similar to our findings, a Chinese cohort study and a Tunisian case-control study showed no significant associations between aMED score and GDM [61, 62]. These inconclusive results may be partly attributed to variations in ethnicity, diagnostic criteria, and socioeconomic backgrounds within the study populations, which can introduce diversity in dietary habits, access to health care, and health outcomes. To our knowledge, there are no data regarding the association between adherence to the Mediterranean diet and GDM risk in the UAE. We found only one study from a neighboring country, aimed to explore sociodemographic and pregnancy-related determinants of Mediterranean diet adherence among Saudi pregnant women (*n* = 774) and found that those who had GDM had a lower tendency to adhere to the Mediterranean diet [63]. However, the number of women with gestational diabetes (*n* = 23, 3%) was very small [63]. The Mediterranean diet has been shown to significantly reduce the risk of GDM in various populations, including non-Mediterranean countries [64]. This suggests that the benefits of the Mediterranean diet for GDM prevention may apply to diverse populations, including women in the UAE. However, further research is needed to draw definitive conclusions about these associations in the UAE’s specific geographic and cultural setting.

### Strength and limitations

To date, this is the first study assessing the association between UPF intake, diet quality, and GDM among pregnant women from the UAE. Our study has several strengths, including a large sample size of pregnant women who are relatively representative of women from the general Emirati population, especially in Al Ain city, as all of the Emirati population has full healthcare insurance, allowing them to access the same level of healthcare in any hospital in all UAE. Nevertheless, more studies covering different parts of the country would be needed for generalizability. Additionally, we were able to account for several potential confounders, including various lifestyle and demographic factors. However, there are lifestyle factors before and during pregnancy that could affect the risk of GDM that were not assessed in this study. For example, we had no information on pre-pregnancy weight status, history of GDM, family history of diabetes, dietary intake before pregnancy, physical activity throughout pregnancy, and psychosocial factors such as anxiety and depression. Further limitations should also be acknowledged, including the reliance on self-reported data and the potential for residual confounding, such as socioeconomic status, sleep, or stress. The FFQ used in this study was not specifically designed to measure UPF intake, which may have led to misclassification, and there is a risk of recall bias or misreporting when using self-reported dietary data, especially among pregnant women who may change their dietary intakes during pregnancy [65]. However, self-reported data has been widely used in epidemiological studies and shows strong consistency and validity in predicting various outcomes [66]. In addition, due to the observational nature of this study, we cannot establish causality, and the study design was not suited to providing detailed evaluations of any underlying mechanisms. Further, dietary data was collected at any time during pregnancy, in some cases after diagnosis of GDM and the data handled in this paper is best described as cross-sectional analysis of dietary intake in relation to GDM. However, we conducted a prospective sensitivity analysis for participants who completed the FFQ before the OGTT test to strengthen the robustness of our findings further. Moreover, we used mean and mode imputations to address missing data points, assuming they were missing at random. However, this approach otherwise may underestimate variability and introduce bias. However, few data points were imputed (13% of the women in both types of the imputation methods); therefore, the imputation is unlikely to have materially impacted the analyses. Considering alternative imputation strategies, such as multiple imputation, in our future studies is warranted to enhance robustness. Lastly, the diagnosis of GDM was made at a single time point, which limited our ability to distinguish between early and late-onset GDM or to account for other factors influencing glucose intolerance, such as GWG or genetic predisposition.

## Conclusions

The results of the present study indicate that higher intake of UPF was associated with an overall negative impact on the diet quality of pregnant women in the UAE. Although no direct associations were observed between UPF intake or aMED and GDM, these findings emphasize the need for further investigation into the effects of diet quality in GDM prevention. Nutritional recommendations and dietary guidance for this population should emphasize nutrient quality, while further research is needed to clarify the health effects of food processing levels during pregnancy.

## Electronic supplementary material

Below is the link to the electronic supplementary material.

Supplementary File 1: Nova groups definitions and classification of FFQ food items

Supplementary File 2: Sensitivity analysis among women who completed the FFQ before the time of GDM screening

## Data Availability

All data generated or analysed during this study are included in this published article and its supplementary information files.

## Abbreviations

aMED: alternate Mediterranean Diet score
BMI: Body Mass Index
CI: Confidence Interval
FFQ: semi-quantitative Food Frequency Questionnaire
GDM: Gestational Diabetes Mellitus
GWG: Gestational Weight Gain
MUFA/SFA: Monounsaturated Fatty Acids to Saturated Fatty Acids ratio
MUFA: Monounsaturated Fatty Acids
OGTT: Oral Glucose Tolerance Test
OR: Odds Ratio
SD: Standard Deviations
SFA: Saturated Fatty Acids
UAE: United Arab Emirates
UPF: Ultra-Processed Food
