# Supplementary material for: Ultra-processed food intake, diet quality, and risk of gestational diabetes mellitus: a cross-sectional analysis from the Mutaba’ah study
Source: Nutr Metab (Lond). 2025 May 26;22:53. doi: 10.1186/s12986-025-00950-z (PMC12105120; doi:10.1186/s12986-025-00950-z)
Supplement: Supplementary file 1 — Nova groups definitions and classification of FFQ food items [file 12986_2025_950_MOESM1_ESM.docx]

**Supplemental File 1**: NOVA groups definitions (1) and classification of the food frequency questionnaire (FFQ) food items. Food items classified as ultra-processed food (UPF) are described in the table below under NOVA group 4.

| **NOVA group** | **Definition (1)** | **FFQ food items** |
| --- | --- | --- |
| **1) Unprocessed or minimally processed foods** | Unprocessed: edible parts of plants (fruits, seeds, leaves, stems, roots, tubers) or of animals (muscle, offals, eggs, milk), and also fungi, algae and water, after separation from nature.  Minimally processed: unprocessed foods altered by industrial processes such as removal of inedible or unwanted parts, drying, crushing, grinding, fractioning, roasting, boiling, pasteurisation, refrigeration, freezing, placing in containers, vacuum packaging, non-alcoholic fermentation, and other methods that do not add salt, sugar, oils or fats or other food substances to the original food. The main aim of these processes is to extend the life of unprocessed foods, enabling their storage for longer use, and, often, to make their preparation easier or more diverse. Infrequently, minimally processed foods contain additives that prolong product duration, protect original properties or prevent proliferation of microorganisms. | Milk/whole//low fat, white cheese, yogurt/regular/low fat, apple, strawberry, banana, Date/dry/fresh, orange, papaya, pineapple, mandarin, grapes, kiwi, fig/dry/fresh, apricot, plum/fresh/dry, peaches/fresh, pear, watermelon, mango, cantaloupe, cherries, guava, pomegranate, chickoo, blueberry, cabbage/fresh/cooked, vegetables mix/cooked, cauliflower/fresh/cooked, eggplant/cooked, hamoose, chickpea/dry, okra/cooked, beans/cooked, foul/lentils/cooked, spinach/cooked, eggs/boiled, chicken or turkey with skin/without skin, beef/lamb or mutton as a main dish (eg. steak, roast,etc), broiled fish, tiger prawn, liver, brown rice/cooked, oats/cooked, quinoa/cooked, burgle/cooked, coffee/nescafe, coffee/Arabic, tea, honey, almond nut, walnut |
| **2) Processed culinary ingredients** | Substances obtained directly from group 1 foods or from nature by industrial processes such as pressing, centrifuging, refining, extracting or mining. Their use is in the preparation, seasoning and cooking of group 1 foods. These products may contain additives that prolong product duration, protect original properties or prevent proliferation of microorganisms. | Labnah/regular/low fat, butter, laban up, cooked green leafy vegetables, white rice with salt, pasta/spaghetti with salt, potatoes/baked/boiled with salt |
| **3) Processed foods** | Products made by adding salt, oil, sugar or other group 2 ingredients to group 1 foods, using preservation methods such as canning and bottling, and, in the case of breads and cheeses, using non-alcoholic fermentation. Processes and ingredients here aim to increase the durability of group 1 foods and make them more enjoyable by modifying or enhancing their sensory qualities. These products may contain additives that prolong product duration, protect original properties or prevent proliferation of microorganisms. | Saman, olives, apricot/canned, peaches/canned, vegetables salad, mutable, hamoose/paste, grape leaves stuffed, gabbage leaves stuffed, zucchini stuffed, eggs/omelet/fried, kuftah, fish curry or saloona, vegetable marag/saloona, magboos, jareesh, margoog, malleh, hareese, mahamer, falafel, sambosa, kebeh Burgle, araeas, fatayer cheese/zatar/labneh/meat, managesh zatar, meat/chicken shawarma, phool sandwich, irani bread, arabic omani halwa, balaleet, faaloodah, khabeesah, mahlabiyyeh, cake/homemade, cashew nuts/salted, peanut/salted, pistachio/salted, seeds/salted |
| **4) Ultra-processed foods** | Formulations of ingredients, mostly of exclusive industrial use, that result from a series of industrial processes (hence ‘ultra-processed’), many requiring sophisticated equipment and technology. Processes enabling the manufacture of ultra-processed foods include the fractioning of whole foods into substances, chemical modifications of these substances, assembly of unmodified and modified food substances using industrial techniques such as extrusion, moulding and pre-frying, frequent application of additives whose function is to make the final product palatable or hyper-palatable (‘cosmetic additives’), and sophisticated packaging, usually with synthetic materials. Ingredients often include sugar, oils and fats, and salt, generally in combination; substances that are sources of energy and nutrients but of no or rare culinary use such as high fructose corn syrup, hydrogenated or interesterified oils, and protein isolates; cosmetic additives such as flavours, flavour enhancers, colours, emulsifiers, sweeteners, thickeners, and anti-foaming, bulking, carbonating, foaming, gelling, and glazing agents; and additives that prolong product duration, protect original properties or prevent proliferation of microorganisms. Processes and ingredients used to manufacture ultra-processed foods are designed to create highly profitable products (low cost ingredients, long shelf-life, emphatic branding), convenient (ready-to-consume) hyper-palatable snacked products liable to displace all other NOVA food groups, notably group 1 foods. | Cream cheese/ whole/low fat, ice cream, margarine, mayonnaise, fried fish, chicken/beef/or lamb burger, vegetable/chicken/beef soup, pizza, cheese croissants, cold breakfast cereal, white bread, khameer/chabab, chapati, brown bread, rusk, barata, french fried potatoes, biscuits/ready-made, racker round/ready-made, coke/pepsi, soft drink, fruit juice, arabic sweets/baklava, aseeda, betheeth, cookies, chocolate, kanafa, lagaimaat, cheese cake, cake/ready-made, rahash |

**References:**

1. Monteiro CA, Cannon G, Levy RB, Moubarac JC, Louzada ML, Rauber F, et al. Ultra-processed foods: what they are and how to identify them. Public Health Nutr. 2019;22(5):936-41.
