# Supplementary material for: Ultra-processed food intake, diet quality, and risk of gestational diabetes mellitus: a cross-sectional analysis from the Mutaba’ah study
Source: Nutr Metab (Lond). 2025 May 26;22:53. doi: 10.1186/s12986-025-00950-z (PMC12105120; doi:10.1186/s12986-025-00950-z)
Supplement: Supplementary file 2 — Sensitivity analysis among women who completed the FFQ before the time of GDM screening [file 12986_2025_950_MOESM2_ESM.docx]

**Supplementary File 2:** Sensitivity analysis among women who completed the Food frequence questionnaire (FFQ) before the time of Gestational Diabetes Mellites (GDM) screening (before week 24 of gestation) (N=321). Table 1. Shows UPF consumption according to GDM diagnosis before week 24 of gestation and Table 2. Shows aMED score index according to GDM diagnosis before week 24 of gestation.

Table 1. UPF consumption according to GDM diagnosis, defined by the NICE-2015 criteria

|  | NICE-2015 criteria** | | |
| --- | --- | --- | --- |
|  | OR | 95 % CI | P value |
| **UPF intake (continuous, servings/day)** |  |  |  |
| Model 1, crude | 0.99 | 0.92-1.07 | 0.83 |
| Model 2, adjusted^1^ | 0.99 | 0.92-1.08 | 0.89 |
| Model 3, adjusted^2^ | 0.99 | 0.92-1.08 | 0.97 |
|  |  |  |  |
| **UPF intake (tertiles, servings/day)** |  |  |  |
| Model 1, crude |  |  |  |
| Tertile 1 | Ref |  |  |
| Tertile 2 | 0.80 | 0.40-1.59 | 0.53 |
| Tertile 3 | 1.06 | 0.56-2.01 | 0.86 |
| Model 2, adjusted^1^ |  |  |  |
| Tertile 1 | Ref |  |  |
| Tertile 2 | 1.02 | 0.48-2.19 | 0.96 |
| Tertile 3 | 1.17 | 0.57-2.40 | 0.66 |
| Model 3, adjusted^2^ |  |  |  |
| Tertile 1 | Ref |  |  |
| Tertile 2 | 1.02 | 0.48-2.19 | 0.96 |
| Tertile 3 | 1.22 | 0.59-2.51 | 0.59 |
|  |  |  |  |
| **UPF food groups** |  |  |  |
| **UPF diary (servings/day)** |  |  |  |
| Model 1, crude | 1.27 | 0.98-1.65 | 0.067 |
| Model 2, adjusted^1^ | 1.25 | 0.95-1.66 | 0.11 |
| Model 3, adjusted^2^ | 1.34 | 0.99-1.81 | 0.058 |
|  |  |  |  |
| **UPF mixed dishes (servings/day)** |  |  |  |
| Model 1, crude | 0.68 | 0.38-1.21 | 0.19 |
| Model 2, adjusted^1^ | 0.56 | 0.27-1.14 | 0.11 |
| Model 3, adjusted^2^ | 0.54 | 0.26-1.14 | 0.11 |
|  |  |  |  |
| **UPF bread (servings/day)** |  |  |  |
| Model 1, crude | 1.13 | 0.91-1.41 | 0.27 |
| Model 2, adjusted^1^ | 1.14 | 0.89-1.45 | 0.31 |
| Model 3, adjusted^2^ | 1.14 | 0.89-1.46 | 0.29 |
|  |  |  |  |
| **UPF sweets (servings/day)** |  |  |  |
| Model 1, crude | 1.07 | 0.76-1.50 | 0.71 |
| Model 2, adjusted^1^ | 1.07 | 0.70-1.62 | 0.75 |
| Model 3, adjusted^2^ | 1.08 | 0.71-1.65 | 0.71 |
|  |  |  |  |
| **UPF beverages (servings/day)** |  |  |  |
| Model 1, crude | 0.78 | 0.56-1.08 | 0.13 |
| Model 2, adjusted^1^ | 0.81 | 0.56-1.17 | 0.26 |
| Model 3, adjusted^2^ | 0.81 | 0.56-1.17 | 0.27 |
|  |  |  |  |

UPF: ultra-processed food, GDM: gestational diabetes mellitus, Ref: reference, NICE-2015: National Institute for Health and Clinical Excellence.^1^Adjusted for maternal age, first trimester BMI, parity, gestational age at the time of the FFQ, education level, occupation status, PA before, husband active smoking. ^2^Adjusted for model two and aMED score. ** N= 292 in the crude model and N= 258 in the adjusted models.

Table 2. aMED score index according to GDM diagnosis, defined by the NICE-2015 criteria

|  | NICE-2015 criteria** | | |
| --- | --- | --- | --- |
|  | OR | 95 % CI | P value |
| **aMED score (continuous)** |  |  |  |
| Model 1, crude | 1.08 | 0.91-1.29 | 0.38 |
| Model 2, adjusted^1^ | 1.06 | 0.87-1.29 | 0.58 |
| Model 3, adjusted^2^ | 1.06 | 0.87-1.29 | 0.59 |
|  |  |  |  |
| **aMED score (tertiles)** |  |  |  |
| Model 1, crude |  |  |  |
| Tertile 1 | Ref |  |  |
| Tertile 2 | 1.32 | 0.73-2.38 | 0.37 |
| Tertile 3 | 1.60 | 0.72-3.56 | 0.25 |
| Model 2, adjusted^1^ |  |  |  |
| Tertile 1 | Ref |  |  |
| Tertile 2 | 1.19 | 0.62-2.32 | 0.59 |
| Tertile 3 | 1.49 | 0.61-3.64 | 0.38 |
| Model 3, adjusted^2^ |  |  |  |
| Tertile 1 | Ref |  |  |
| Tertile 2 | 1.19 | 0.62-2.32 | 0.59 |
| Tertile 3 | 1.49 | 0.60-3.67 | 0.39 |
|  |  |  |  |
| **aMED food groups** |  |  |  |
| **Vegetable (100 g/d)** |  |  |  |
| Model 1, crude | 0.99 | 0.77-1.28 | 0.95 |
| Model 2, adjusted^1^ | 0.89 | 0.67-1.18 | 0.39 |
| Model 3, adjusted^2^ | 0.88 | 0.66-1.17 | 0.39 |
|  |  |  |  |
| **Fruits (100 g/d)** |  |  |  |
| Model 1, crude | 0.97 | 0.89-1.06 | 0.54 |
| Model 2, adjusted^1^ | 0.96 | 0.87-1.06 | 0.38 |
| Model 3, adjusted^2^ | 0.95 | 0.85-1.05 | 0.32 |
|  |  |  |  |
| **Nuts (30 g/d)** |  |  |  |
| Model 1, crude | 1.16 | 0.94-1.42 | 0.18 |
| Model 2, adjusted^1^ | 1.11 | 0.88-1.39 | 0.39 |
| Model 3, adjusted^2^ | 1.11 | 0.87-1.41 | 0.39 |
|  |  |  |  |
| **Whole grain (30 g/d)** |  |  |  |
| Model 1, crude | 0.97 | 0.74-1.28 | 0.85 |
| Model 2, adjusted^1^ | 0.87 | 0.64-1.19 | 0.40 |
| Model 3, adjusted^2^ | 0.87 | 0.63-1.21 | 0.40 |
|  |  |  |  |
| **Legumes (100 g/d)** |  |  |  |
| Model 1, crude | 0.72 | 0.33-1.57 | 0.41 |
| Model 2, adjusted^1^ | 0.56 | 0.21-1.49 | 0.25 |
| Model 3, adjusted^2^ | 0.55 | 0.21-1.48 | 0.24 |
|  |  |  |  |
| **Red meat (100 g/d)** |  |  |  |
| Model 1, crude | 0.56 | 0.18-1.77 | 0.33 |
| Model 2, adjusted^1^ | 0.42 | 0.12-1.50 | 0.18 |
| Model 3, adjusted^2^ | 0.41 | 0.12-1.48 | 0.17 |
|  |  |  |  |
| **Fish (100 g/d)** |  |  |  |
| Model 1, crude | 1.18 | 0.59-2.39 | 0.64 |
| Model 2, adjusted^1^ | 1.36 | 0.62-2.97 | 0.45 |
| Model 3, adjusted^2^ | 1.36 | 0.62-2.98 | 0.44 |
|  |  |  |  |
| **MUFA/SFA ratio (g/d)** |  |  |  |
| Model 1, crude | 1.17 | 0.79-1.73 | 0.42 |
| Model 2, adjusted^1^ | 1.07 | 0.69-1.64 | 0.77 |
| Model 3, adjusted^2^ | 1.06 | 0.69-1.65 | 0.78 |

aMED: alternate Mediterranean Diet score, GDM: gestational diabetes mellitus, Ref: reference, NICE-2015: National Institute for Health and Clinical Excellence.^1^Adjusted for maternal age, first trimester BMI, parity, gestational age at the time of the FFQ, education level, occupation status, PA before, husband active smoking. ^2^Adjusted for model two and UPF intake. ** N= 292 in the crude model and N= 258 in the adjusted models.
